# Ocular development after highly effective modulator treatment early in life

**DOI:** 10.3389/fphar.2023.1265138

**Published:** 2023-09-19

**Authors:** Yimin Zhu, Danni Li, Felisa Reyes-Ortega, Holly R. Chinnery, Elena K. Schneider-Futschik

**Affiliations:** ^1^ Department of Biochemistry and Pharmacology, School of Biomedical Sciences, Faculty of Medicine, Dentistry and Health Sciences, The University of Melbourne, Parkville, VIC, Australia; ^2^ Department of Ophthalmology, Maimonides Biomedical Research Institute of Cordoba (IMIBIC), Reina Sofia University Hospital and University of Cordoba, Cordoba, Spain; ^3^ Department of Optometry and Vision Sciences, The University of Melbourne, Parkville, VIC, Australia

**Keywords:** cystic fibrosis, ocular development, CFTR modulator, catarat, eye malformations

## Abstract

Highly effective cystic fibrosis (CF) transmembrane conductance regulator (CFTR) modulator therapies (HEMT), including elexacaftor-tezacaftor-ivacaftor, correct the underlying molecular defect causing CF. HEMT decreases general symptom burden by improving clinical metrics and quality of life for most people with CF (PwCF) with eligible CFTR variants. This has resulted in more pregnancies in women living with CF. All HEMT are known to be able pass through the placenta and into breast milk in mothers who continue on this therapy while pregnant and breast feeding. Toxicity studies of HEMT in young rats demonstrated infant cataracts, and case reports have reported the presence of congenital cataracts in early life exposure to HEMT. This article reviews the evidence for how HEMT influences the dynamic and interdependent processes of healthy and abnormal lens development in the context of HEMT exposure during pregnancy and breastfeeding, and raises questions that remain unanswered.

## Introduction

Since the approval of the highly effective modulator therapies (HEMT) targeting the cystic fibrosis transmembrane conductance regulator (CFTR) protein, the use of HEMT have been crucial alongside the supplementary symptomatic treatments for cystic fibrosis (CF) (Bell et al., 2020a). HEMT are small molecules designed to target the specific underlying gene defect, with the ability to modulate CFTR protein synthesis, trafficking, and functioning, ultimately restoring the CFTR channels residing on various epithelial cell surfaces. From the early first-generation modulator ivacaftor as a monotherapy to the progression of ivacaftor combination therapies including ivacaftor-lumacaftor and ivacaftor-tezacaftor, HEMT have displayed safety and efficacy in preclinical and clinical trials (Cai et al., 2011). In recent years, triple combination of elexacaftor-tezacaftor and ivacaftor (ETI), as well as the ongoing phase II trials involving vanzacaftor-tezacaftor-deutivacaftor combination have been the main focus to increase treatment options for CF patients (Ghelani and Schneider-Futschik, 2020).

Along with the progression in drug development, some adverse events have been noticed through preclinical and clinical trials involving HEMT. The use of ivacaftor in juvenile rodents and young patients is associated with non-congenital cataracts and this has been observed in several ivacaftor—combination therapies (FDA, 2021). Due to the ongoing lens development in humans from birth to adulthood, the influence of chemicals including drugs is hypothesised as a potential explanation for the ocular defects observed in clinical studies (Van Cruchten et al., 2017).

**FIGURE 1:**
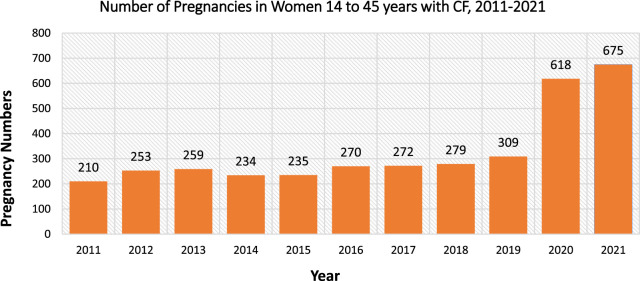
United States cystic fibrosis registry data on the number of pregnancies per year in women with cystic fibrosis at 14–45 years from 2011 to 2021. Data obtained from Cystic Fibrosis Foundation Patient Registry 2021 Annual Data Report (Foundation, 2022).

As a result of the novel ETI therapy, more women with CF (wwCF) are reaching childbearing age, doubling the number of pregnancies from 2019 to 2021 (Figure 1) (Foundation, 2022). Since pregnant patients and babies are typically excluded from clinical trials, all safety data to date stems from case reports/series. Recently, clinical case series provided evidence of an increased risk of developing congenital cataracts in newborns after *in utero* exposure to ETI (Jain et al., 2022). The embryonic and fetal stages are particularly vulnerable to exogenous agents, making drug use during these stages highly likely to influence ocular development (Sachdeva et al., 2009; Li D and Schneider-Futschik, 2023).

## Cystic fibrosis

CF is an autosomal recessive condition resulting from variations in the CFTR gene, which encodes for the CFTR ion channel that conducts chloride and bicarbonate transport (Kerem et al., 1989; Rang et al., 2020). Since CFTR is abundant on epithelial cells, CF symptoms can manifest in various organs e.g., lung, liver, and pancreas (Rang et al., 2020; Li D and Schneider-Futschik, 2023). Therefore, despite lung symptoms being the primary cause of mortality, CF is considered a multi-organ disease (Bell et al., 2020a).

The CFTR gene has over 2,000 possible variants that can be categorised into 6 classes (Rang et al., 2020). These classes provide the basis for “mutation-specific therapies” which aim to use the same therapeutic strategy for mutations categorized in the same class (Amaral and Kunzelmann, 2007). This propelled the discovery of highly effective modulator therapies (HEMT) through high throughput screening of compounds tested on specific CFTR mutation expressing cell lines (Lopes-Pacheco, 2019). Currently, HEMT can be categorised into either correctors or potentiators which both act to modulate the CFTR protein directly to target the underlying gene defect in CF. Correctors can rescue the defect trafficking of CFTR onto the cell surface and can be used in combination with potentiators that maintains the opening time and opening probability of CFTR channels (Ramsey et al., 2011; Bell et al., 2020a; Ghelani and Schneider-Futschik, 2020).

## HEMT

Ivacaftor is the first CFTR potentiator approved for patients ≥3 months old with at least one gating variation e.g., G551D class III, which is present in around 4% of the CF patient population (Ghelani and Schneider-Futschik, 2020). In 2023, after the completion of a phase 3 ARRIVAL clinical trial that evaluated ivacaftor use in infants under 24 months old, the United States Food and Drug Administration expanded the use of ivacaftor for infants up to 1 month old that are carrying at least one mutation responsive to ivacaftor (FDA, 2023a). The ARRIVAL study revealed that the pharmacokinetics of ivacaftor in children aged 12–24 months were similar in distribution to older children and adults, and the adverse events were unlikely to be associated with ivacaftor use (Rosenfeld et al., 2018; Davies et al., 2021). In addition, the observed clinical efficacy through biomarkers of CFTR function overall supports the use of ivacaftor to slow or prevent CF progression in newborns and young patients.

Since ivacaftor is limited to mostly class III and rarer mutations, correctors that target the F508del mutation which would be available for 90% of CF patients were developed to benefit the wider population (Cai et al., 2011). Lumacaftor-ivacaftor combination as a corrector-potentiator combination acts to first rescue the defective processing of CFTR protein in the endoplasmic reticulum and then enhance the gating activity of CFTR proteins with F508del mutations (Van Goor et al., 2011). *In vitro* studies highlighted the combination effect of lumacaftor-ivacaftor inducing greater CFTR-mediated chloride transport than lumacaftor alone, and this similar observation of higher clinical benefit compared to ivacaftor alone was replicated in studies on homozygous F508del patients (Van Goor et al., 2011; Wainwright et al., 2015). Based on the successful phase 3 study in 2021, FDA has approved the use of lumacaftor-ivacaftor for children over 1 year old with the homozygous F508del mutation (FDA, 2022a; Rayment et al., 2022). Similarly, tezacaftor-ivacaftor is a synergistic corrector-potentiator combination therapy that is available for children aged 6 years old who have at least one mutation responsive to therapy (FDA, 2022b). Compared to the lumacaftor-ivacaftor combination, tezacaftor-ivacaftor does not interact with CYP3A4 enzymes which reduces potential drug interaction and metabolising issues but maintains its clinical efficacy (Donaldson et al., 2018; Schneider, 2018).

In 2019 (US) and 2021 (Australia), the first triple combination therapy involving elexacaftor-tezacaftor-ivacaftor was approved for patients aged over 12 years old. Elexacaftor is a next-generation modulator that is proposed to act as both corrector and potentiator (Shaughnessy et al., 2021). In phase 3 studies, clinical end points of FEV1 were superior to the tezacaftor-ivacaftor combination with fewer adverse events (Heijerman et al., 2019; Middleton et al., 2019). Following on, clinical trials have confirmed the safety profile, efficacy and pharmacokinetics of ETI in patients aged 2–5 years old (Rayment et al., 2022; FDA, 2023b; Goralski et al., 2023). To date, the FDA has announced the availability of ETI for children from 2 years old with an F508del allele or any mutation responsive to therapy.

Apart from the approved modulators, there is ongoing development involving a novel triple combination of vanzacaftor-tezacaftor-deutivacaftor that has undergone phase 2 clinical trials in patients over 18 years. It is aimed to achieve once daily dosing for patients who have at least one copy of the F508del mutation and exceed the clinical benefit of ETI which is currently the benchmark therapy for F508del mutations (Uluer et al., 2023). Undoubtedly, if it displays superior efficacy and tolerable safety profiles, clinical trials will proceed to young patients since it is hoped that HEMT treatment options can extend to younger age groups to control manifestations of CF symptoms early.

## Cystic fibrosis and pregnancy

Symptoms of CF extend to the reproductive system (Kaplan et al., 1968). Since the 1970 s, wwCF were described having lower fertility rates compared with healthy women (Kopito et al., 1973; Shteinberg et al., 2019). Dehydrated cervical mucus and disrupted pH balance can disrupt sperm viability for successful fertilisation (Kopito et al., 1973; Chan et al., 2002; Wang et al., 2003; Hughan et al., 2019). Furthermore, CFTR has been identified in the hypothalamus of both rats and humans where the regulation of homeostatic reproductive functions such as gonadotrophin-releasing hormone production may consequently be disrupted in CF patients (Mulberg et al., 1998; Hughan et al., 2019; Qiu et al., 2020).

Apart from indirectly enhancing health status, CFTR modulators may also directly restore fertility by correcting CFTR channel located in the cervix to decrease cervical mucus tenacity (Jones and Walshaw, 2015). Jones et al. support the hypothesis by displaying restored fertility in women despite no significant changes in the overall health status (Jones and Walshaw, 2015). Additionally, hysteroscopic images of the vagina highlights the clearance of mucus plugs after starting ivacaftor, further emphasising that restoration of CFTR localised in the reproductive system can improve fertility (Jain and Taylor-Cousar, 2021). The approval of ETI consequently escalated the number of pregnancies due to the high drug efficacy and mild adverse events with wide population suitability (Figure 1) (Middleton et al., 2019). Specifically, ETI have been proposed to have the potential to increase conception, even in patients with a history of subfertility (O'Connor et al., 2021).

## Safety of HEMT in pregnancy

It is well established in preclinical and clinical studies that all components of HEMT will pass through the placenta which raises concern for the effect it may have on fetal development (Trimble et al., 2018; FDA, 2021; Qiu et al., 2021; Collins et al., 2022; Jain et al., 2022). As more pregnancies are reported under ETI treatment, this paragraph focuses on ETI.

In animal reproductive models, the use of ETI during the organogenesis period at normal doses is safe, and at maternally toxic doses, ETI does not cause significant developmental defects (FDA, 2021). Similarly, in clinical cases reported, first-generation CFTR modulatory drugs are well tolerated, and emerging case reports of ETI use indicates that most maternal and fetal complications are unrelated to its use (Table 1) (Nash et al., 2020; Taylor-Cousar, 2020). Moreover, a withdrawal effect initially proposed by Trimble et al. is observed after stopping ETI treatment in which the patient’s pulmonary function deteriorates significantly (Trimble and Donaldson, 2018). The impact of the withdrawal effect has prompted many wwCF to re-initiate therapy (Trimble and Donaldson, 2018; Nash et al., 2020; Taylor-Cousar and Jain, 2021). Conversely, despite some promising and increasing use of ETI reported, the government’s drug for pregnancy categorisation systems have indicated that ultimately, the lack of sufficient clinical studies of ETI use during pregnancy makes it difficult to establish certainty in its safety. Therefore, observations of complications from individual case reports should be prioritized for evaluation.

**TABLE 1:** ETI use during pregnancy and associated complications in mothers and fetuses.

Case reports	Pregnancies	Miscarriage/Prematurity	Fetal complications	Maternal complication	
			Unknown/not related to ETI	Related to ETI	Unknown/not related to ETI
Taylor-Cousar and Jain (2021)	45	4 (1 was of unknown relatedness to ETI use) and 5 (unrelated to maternal ETI use)	5 events in 3 infants/15 events in 15 infants	1 event in 1 woman *	2 events in 2 women/28 events in 21 women
Collins et al. (2022)	3		cephalohematoma and newborn jaundice due to complicated delivery	Maternal cholecystitis [same case as Taylor-Cousar and Jain (2021)*]	
Jain and Taylor-Cousar (2021)	1		possible small fetal pericardial effusion but insignificant		
Burn et al. (2022)	5	1 miscarriage	3 require NICU admission, 2 were transient tachypnoea		2 hypertensive disorders of pregnancy in 2 women
Balmpouzis et al. (2022)	1				2 complications resolved
Chamagne et al. (2022)	1	1 prematurity due to rupture of membrane	transitory respiratory distress syndrome		1 pulmonary exacerbation resolved and no hospitalisation required
Cimino et al. (2023)	1				
Fortner et al. (2021)	1				
Kendle et al. (2021)	5			Transaminitis suspected due to ETI use	2 CF exacerbation in 1 woman
Sionidou et al. (2023)	1				
Balmpouzis et al. (2022)	1				Complication arised after 31st week, required hospitalisation
Gómez-Montes et al. (2023)	1		Meconium ileus due to CF resolved after ETI initiation		
Szentpetery et al. (2022)	1		Meconium ileus due to CF resolved after ETI initiation		Gestational hypertension

### HEMT and the development of cataracts

Non-congenital cataract formation with ivacaftor treatment has been reported in preclinical rat studies and in children receiving both ivacaftor monotherapy and ivacaftor combined with other CFTR modulators like lumacaftor. (Talamo Guevara and McColley, 2017). However, the exact pathophysiology or critical periods of exposure of ivacaftor-associated cataracts remains uncertain. (Kramer and Clancy, 2016).

Juvenile rats that were dosed from post-natal days 7–35 developed cataracts even at doses 0.1 times the maximum recommended human dose (FDA, 2023a). Since this was only observed in the juvenile age group, it potentiates that ivacaftor may modulate early ocular developmental milestones which are subsequently observed in clinical studies. A number of studies associated with the manufacturer of HEMT found that 4.17% of CF patients between 2 and 6 years old developed cortical cataracts within 84 weeks of starting ivacaftor and 0.57% of CF patients 12 years and older developed subcapsular cataracts within 96 weeks of starting treatment. (R/0106 K.-E. H. C,). Similarly, 1.72% of CF patients aged between 6 and 11 developed unspecified forms of cataracts within 24 weeks of starting lumacaftor combined with ivacaftor, and 2 out of 130 participants aged 6 years or older undertaking combination of ivacaftor-tezacaftor experienced cataract (Incorporated, 2017a; European Medicines Agency, 2020). Although in the recent clinical trials involving young patients under 1 year old, ivacaftor did not cause cataract development, 1 case of lenticular opacity was observed in children from 2 to 5 years old under ETI combination regime (Rosenfeld et al., 2018; Davies et al., 2021; Goralski et al., 2023). These concerning findings prompted the FDA to suggest the conductance of ophthalmologic examination before and following ivacaftor monotherapy or combination modulator treatments in clinical settings. Given the anticipated rise in the number of younger patients who will be on modulator therapy in the future, this is an urgent research priority as the development of cataracts and risk of blindness will be devastating for these children and should not be accepted as trading one life-changing disease with another.

A recent clinical series in which 3 out of 23 infants were diagnosed with congenital cataracts after being born to mothers who were taking ETI highlights a potential risk of ETI use during pregnancy (Table 2) (Jain et al., 2022). In all cases of cataracts, mothers carrying at least one copy of the F508del mutation had been on ETI before their conception and continued treatment throughout pregnancy. There were no known risk factors and no family history of cataracts, suggesting that ETI may be the main contributor to this defect. Previous cases of non-congenital cataracts of unknown pathophysiology associated with ivacaftor use in juvenile animals and pediatric patients have already raised concern about the possible ocular influences ivacaftor may have (Table 2). Despite subsequent recommendations to assess for cataracts after *in utero* exposure to ivacaftor-containing therapies not revealing any clinical cases of cataracts in infants, there are limited cases studies and formal ophthalmic tests conducted to draw a definite conclusion (Nash et al., 2020). Currently, the three cases of cataracts are < 3 mm which is classified as visually insignificant thus they do not require surgical intervention at this stage. However, this study brings awareness to the potential adverse effect of ETI-related cataract development in newborns since no animal toxicity study has been conducted using ETI as the three-drug combination therapy concomitantly. Since recent reports of babies whose mothers were taking ETI during pregnancy are becoming increasingly common, there is the need to further validate the safety of ETI during these critical developmental periods.

**TABLE 2:** Cataract occurrence and the use of CFTR modulator drugs.

Drug use in animal/CF children	Presence of cataract	Incidence	Type of cataract
Ivacaftor FDA (2023a)	Rat dosed from post-natal day 7–35 at dose of 0.1–0.8 times of the MRHD	All doses	Unknown
Ivacaftor Incorporated (2017b)	CF patients between 2–6 years old within 84 weeks of starting ivacaftor 75 mg	1/24 (4.17%)	cortical
Ivacaftor + Lumacaftor Incorporated (2017a)	CF patients 12 years and older developed within 96 weeks of starting drug	1/176 (0.57%)	subcapsular
Ivacaftor + Lumacaftor Incorporated (2017c)	CF patients at age of 6–11 years within 24 weeks of starting drug	1/58 (1.72%)	Not specified
Ivacaftor + Tezacaftor FDA (2022b)	Yes, but not specified	Unknown	Not specified
Ivacaftor + Tezacaftor European Medicines Agency (2020)	2 cases of cataracts in patients aged 6 years and older	2/130 (1.54%)	Not specified
ETI use Goralski et al. (2023)	1 case of mild lenticular opacity in patients aged 2 to 5	1/75 (1.33%)	

Drug use in pregnant women	Presence of cataract	Incidence	Type of cataract
Taylor-Cousar and Jain (2021) ETI use	None observed in infants after ETI use in pregnancy (2 formal assessments pending)	0/29	
Sionidou et al. (2023) ETI use	No sign of cataract in infant after ETI use in pregnancy	0/1	
Chamagne et al. (2022) ETI use	No evidence of congenital malformation in infants after ETI use in pregnancy	0/2	
Fortner et al. (2021) ETI use	Normal ophthalmologic exam at 2 months in infants after ETI use in pregnancy	0/1	
Jain et al. (2022) ETI use	3 cases of bilateral cataract after ETI use in pregnancy	3/23 (13.04%)	2 nuclear, 1 cortical

### Lens development

Eye development, regulated by the transcriptional factor Pax6, begins with the protrusion of optic vesicles from the diencephalon by gestational day (GD) 25 (Chow and Lang, 2001). The optic vesicle contacts the surface ectoderm layer and invaginates into the midsagittal plane, resulting in the ectodermal cells forming the lens and the optic vesicle forming the retina (Santana and Waiswo, 2011). Simultaneously, the basement membrane of the surface ectodermal cells are positioned as the outer surface and become the lens capsule to encapsulate the detached lens vesicle by gestational week (GW) 6 (Danysh and Duncan, 2009; O'Rahilly, 1975). This transparent membrane thickens via deposition of matrix from lens cells to function as a key anchor for ciliary zonules that control lens accommodation, to regulate the passage of metabolic components into the avascular lens and act as a barrier against infectious agents (Danysh and Duncan, 2009). Lens capsule formation is an important developmental stage, as prior to this stage, the lens is vulnerable to infectious agents. In a study on *in utero* exposure to rubella virus after the human lens capsule has been formed at GW6, Karkinen-Jääskeläinen et al. observed normal eye morphogenesis, which contrasted to the lens fibre degeneration that occurred when the exposure to rubella was at GW4-5, before lens capsule formation was complete (Karkinen-Jääskeläinen et al., 1975). Thus, this highlights that a well-developed lens capsule may mitigate damaging factors during embryogenesis.

**FIGURE 2:**
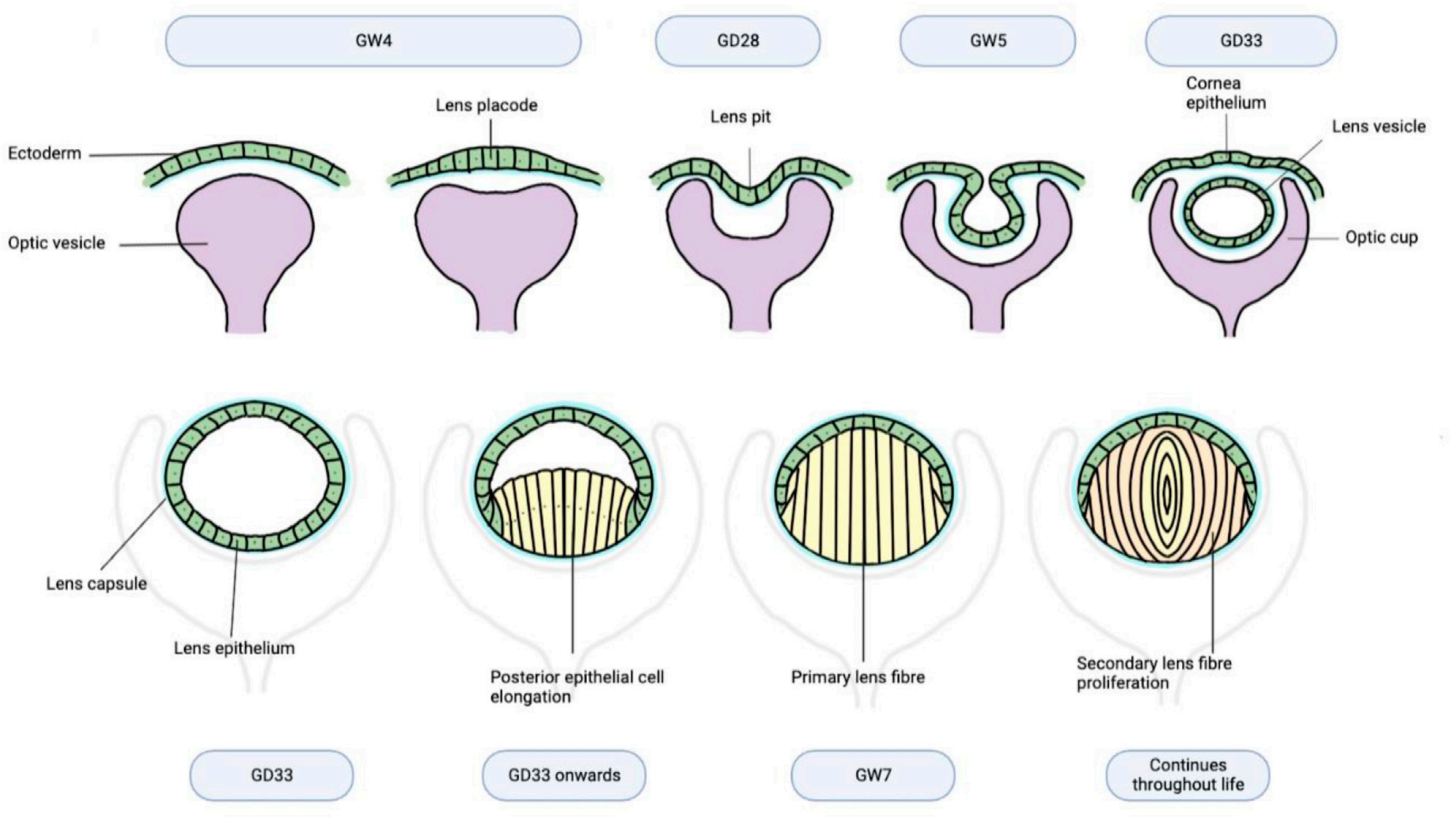
Human lens morphogenesis during gestation.

Primary lens fibre cells differentiate from lens epithelial cells and elongate to form the embryonic nucleus within the lens vesicle by the end of GW7 (Figure 2) (Van Cruchten et al., 2017). Subsequently, differentiated secondary lens fibres form concentric layers upon the original fibre mass and meet to form Y-shaped sutures visible at the end of the 3rd gestational month (O'Rahilly, 1975). Critically, to maintain lens transparency, central lens fibre cells will accumulate crystallin proteins and undergo constitutive de-nucleation and degradation of organelles. This process continues within the structurally compact lens and in the context of uniform proliferation of secondary fibre cells throughout life (McAvoy et al., 1999).

Despite the adult lens being completely avascular, during development, it is supplied by a dense network of transient vessels that surround the lens capsule. To support lens growth by providing nutrients and oxygen, the anterior pupillary membrane and *tunica vasculosa lentis* from the hyaloid vasculature envelop the anterior and posterior hemispheres of the lens respectively by GW9 (Wang et al., 2019). The importance of hyaloid vasculature is supported by observations of smaller lenses and nuclear cataracts in mouse studies where the hyaloid capillaries were not formed in fetal life (Garcia et al., 2009). However, normal regression of fetal vasculatures involving hyalocytes and macrophages is equally crucial since the persistence of capillaries after birth will lead to congenital ocular anomalies such as persistent pupillary membranes (Zhu et al., 2000; Wang et al., 2019).

## Rat eye development

To gain preclinical insight from animal toxicity studies on eye development, it is essential to identify the differences between rat models and humans. Structurally, rat eyes are a quarter the size of humans’, but the ratio of their lens thickness to axial length is around four-fold greater than humans’ (Shibuya et al., 2015). Zonular fibres that originate from the ciliary muscle and attach to the lens capsule are present in both species (Van Cruchten et al., 2017). Yet due to the poor development of this ciliary musculature in and the apparent lack of accommodation needs in rat eyes, the functional significance of the zonular fibres to modulate lens shape is less clear in rat eyes (Parker and Picut, 2016). Despite these differences, the embryogenesis process in rats is similar to humans, where the invagination of the ventral forebrain from GD11 begins the process of optic vesicle formation (Parker and Picut, 2016). However, in the short 21–23 days of gestational period, rat eye morphogenesis is significantly less developed at birth as seen in the eyelids, iris, and hyaloid vasculatures (Table 3).

**TABLE 3:** Comparison between human and rat on the developmental stages of the eye.

	Human	Rat
Gastrulation	GD17 Van Cruchten et al. (2017)	GD8.5-9.5
Optic vesicle forms	GD25 Van Cruchten et al. (2017)	GD11 Parker and Picut (2016)
Optic cups forms	GD28 Van Cruchten et al. (2017)	GD13 Braekevelt and Hollenberg (1970)
Iris and ciliary body	Start to develop at GD30–35, iris fully develop by GW7, ciliary body developed by 5 months of gestation Van Cruchten et al. (2017)	Underdeveloped at birth, histo-morphologically mature at post-natal day (PND) 21 and PND14 respectively Vrolyk et al. (2018)
Hyaloid system forms	GW4-5 Van Cruchten et al. (2017)	GD13 Braekevelt and Hollenberg (1970)
Hyaloid system regression	Start to regress at GW 17 Danysh and Duncan (2009) completely regresses around GW 35–36 Vrolyk et al. (2018)	Completely regresses around PND21 Van Cruchten et al. (2017)
Lens vesicle formation	GD33 Strömland et al. (1991)	GD14 Braekevelt and Hollenberg (1970)
Primary lens fibres proliferation	Complete by GW7 Van Cruchten et al. (2017)	GD15 Braekevelt and Hollenberg (1970)
Secondary lens fibres proliferation	GW6 Strömland et al. (1991)	
Lens capsule	GW5 O'Rahilly (1975)	GD13 Parmigiani and McAvoy (1984)
Eyelid form but fused	GW10 Van Cruchten et al. (2017)	GD18 Parker and Picut (2016)
Eyelid separation	GW26—birth Van Cruchten et al. (2017)	PND12-14 Parker and Picut (2016)
Optic nerve	GD47-48 Van Cruchten et al. (2017)	GD14 Van Cruchten et al. (2017)
Axon myelination	Begins from 7th month of gestation and finishes at up to 1 month after birth Van Cruchten et al. (2017)	PND8 and finishes between PND14-16 Van Cruchten et al. (2017)

## Childhood cataract

Cataracts are opacifications within the lens that can be classified based on their location and time of onset (Bell et al., 2020b). Childhood cataracts of both congenital and juvenile onset have a rarer incidence of 1.8–3.6/10,000 per year compared to age-related cataracts (Sheeladevi et al., 2016). The aetiology of childhood cataracts is diverse and, in some cases, the cause is unknown (Lam et al., 2015; Sheeladevi et al., 2016). While hereditary genetic conditions account for a majority of congenital bilateral cataracts, environmental factors including metabolic disorders and trauma can also lead to cataracts at birth (Bell et al., 2020b). Additionally, intrauterine influences including maternal infections, radiation exposure and maternal drug use may also increase the risks (Churchill and Graw, 2011).

### Drug-induced cataract

**FIGURE 3:**
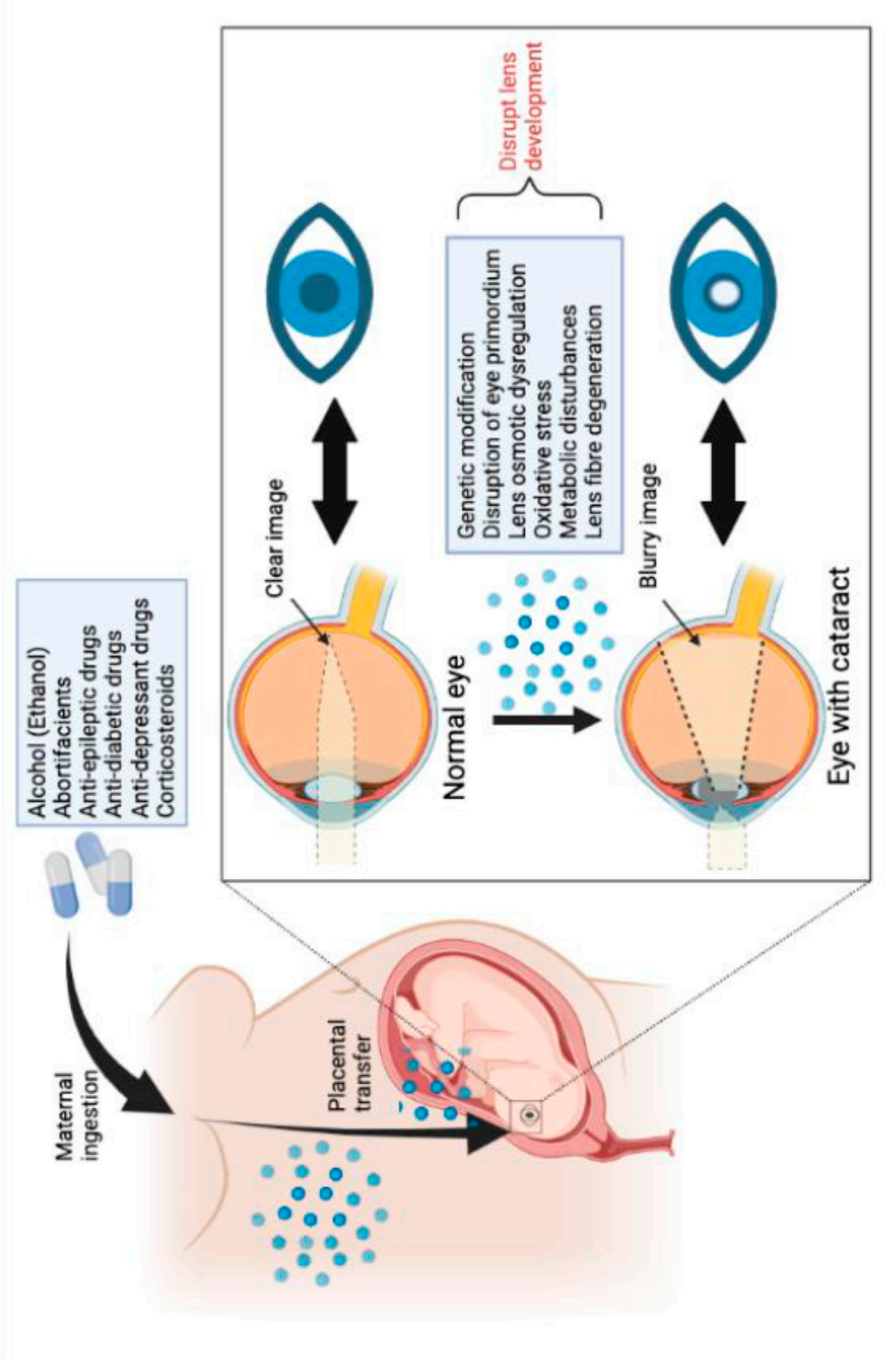
Drug induced development of cataracts via fetal drug transfer.

Eye malformations can occur during the critical embryonic stage due to *in utero* exposure to drugs (Figure 3). (Strömland et al., 1991). Ethanol is a known teratogenic substance shown to disrupt the early induction of the eye primordium via altering gene expression (Miles, 1995). Cook et al. suggest that major teratogenic effects are only observed in the mouse fetal eye when ethanol is administered before GD8 Once the fetal eye is exposed, the altered eye morphogenesis is irreversible (Cook et al., 1987). In past aetiology studies, maternal ingestion of abortifacients, anti-epileptic, anti-diabetic drugs, and corticosteroids are proposed to be associated with congenital cataracts (Angra, 1987; Strömland et al., 1991). Despite the specific mechanism of action being unknown, apart from genetic modification, other possible pathological mechanisms induced by drug exposure include lens osmotic dysregulation, oxidative stress, and metabolic disturbances (Jobling and Augusteyn, 2002). Research by Jobling et al. hypothesises that steroid exposure causes cataract by altering growth factor expression to signal lens epithelial cells to migrate and aggregate at the posterior pole of lens (Jobling and Augusteyn, 2002). Whilst in exposure to anti-depressant drug (TP0446131), the observations of lens fibre degeneration were proposed to be due to TP0446131-related disturbance of cholesterol biosynthesis that was essential for lens fibre saturation (Iwasaki et al., 2020). Ultimately, various mechanisms can disrupt the micro-environment of the lens biochemistry, leading to abnormal light absorption or light scattering of the eye that can progresses to vision-impairing cataract (Bell et al., 2020b).

## Conclusion

Emerging data from case reports and case series (paired with some animal reproduction data) of the use of HEMT during pregnancy provides encouragement about drug safety during pregnancy and breastfeeding. However, due to reports of acute deterioration in health following cessation of HEMT risks to the mother’s health due to discontinuation of HEMT must be weighed carefully against the unknown risks to the fetus. The development of non-congenital cataracts in juvenile rats and published case reports in paediatric patients highlight the need for infant ophthalmologic exams. Thus, a better understanding of the potential risks of HEMT during early life exposure is urgently needed.

## Abbreviations

CF, Cystic fibrosis; CFTR, Cystic fibrosis transmembrane conductance regulator; HEMT, Highly effective modulator treatment; FEV1, Forced expiratory volume in 1 s; wwCF, Women with CF; ETI, Elexacaftor tezacaftor ivacaftor.
